# Intrinsic control of axon regeneration

**DOI:** 10.1016/S1674-8301(10)60002-4

**Published:** 2010-01

**Authors:** Zhigang He

**Affiliations:** F.M. Kirby Neurobiology Center, Children's Hospital, and Department of Neurology, Harvard Medical School, 300 Longwood Avenue, Boston, MA 02115, USA

## Abstract

Spinal cord injury disrupts the connections between the brain and spinal cord, often resulting in the loss of sensory and motor function below the lesion site. The most important reason for such permanent functional deficits is the failure of injured axons to regenerate after injury. In principle, the functional recovery could be achieved by two forms of axonal regrowth: the regeneration of lesioned axons which will reconnect with their original targets and the sprouting of spared axons that form new circuits and compensate for the lost function. Our recent studies reveal the activity of the mammalian target of rapamycin (mTOR) pathway, a major regulator of new protein synthesis, as a critical determinant of axon regrowth in the adult retinal ganglion neurons[1]. In this review, I summarize current understanding of the cellular and molecular mechanisms that control the intrinsic regenerative ability of mature neurons.

## Two types of axon re-growth for functional recovery after CNS injury.

Injury to the mammalian adult central nervous system (CNS) often results in functional deficits, largely owing to the limited regenerative and repairing capabilities. In the case of spinal cord injury, the disruption of axonal tracts that convey ascending sensory and descending motor information could lead to pronounced and persistent sensorimotor dysfunctions in the body parts below the lesion sites. Although partial spontaneous functional recovery occurs in the patients and animal models at the neonatal stages, this declines in the adult. Presumably, rebuilding the functional circuits may result from two types of axon regrowth: ① true regenerative growth of injured axons and ② compensatory sprouting from spared fibers. While regenerative growth occurs rarely in the adult CNS, compensatory sprouting of the same or different types of axons may form new circuits across the lesion sites and compensate for the function lost as the result of injury. Thus, ideal repair strategies could be to promote these two different forms of axon regrowth for optimal functional recovery.

## Importance of the loss of intrinsic growth ability in regeneration failure

In contrast to robust axon growth during development, both regenerative growth and compensatory sprouting in the adult CNS are very limited and abortive. Many studies in the past decades have been largely focused on characterizing environmental inhibitory molecules in the adult CNS[2]–[7]. Several myelin associated molecules and chondroitin sulfate proteoglycans (CSPGs) in the glial scar have been implicated as inhibitors of axon regeneration[2]–[7]. A few critical signaling molecules mediating these inhibitory activities have also been identified. However, when blocking such inhibitory activities by either genetic or pharmacological means, only limited axon regeneration is observed in experimental spinal cord injury models[3],[6],[7]. Furthermore, despite demonstrations that some injured axons are able to regrow into the permissive grafts, the majority of adult neurons fail to regenerate axons when provided with permissive substrate[8]. Together, these studies suggest that removing inhibitory activities is not sufficient to allow the majority of injured CNS axons to regenerate, pointing to the importance of understanding the mechanisms controlling the intrinsic axon growth/regenerative abilities of neurons.

## Growth factor-dependent axon growth during development

A potentially useful approach to understand the intrinsic mechanisms of axon regeneration is to study how robust axon growth in immature neurons during development is achieved. Many of these studies involve neurotrophin-dependent axon growth of peripheral neurons. For example, by using specific chemical inhibitors, Liu and Snider[9] showed that nerve growth facton (NGF)-dependent axon growth from E13 sensory neurons require the activation of Erk kinase (MEK)-extracellular signal-regulated kinase (ERK), phosphatidylinositol-3 kinase (PI3-K), but not janus kinase (JAK) signaling. Interestingly, these pathways mediate distinct aspects of axon growth. For example, activated Raf-1 causes axon lengthening comparable to NGF, while active Akt increases axon caliber and branching[10]. In the case of CNS neurons, previous work suggests that peptide-based growth factors are clearly important for stimulating rapid axon growth, although they may not be sufficient. For example, Ben Barres' lab demonstrated that the combination of neuronal activity (or cAMP) and growth factors is needed to promote the survival and axon growth of cultured retinal ganglion neurons (RGCs)[11]. Recent studies showed that insulin-like grawth factor (IGF) could promote axon growth from cultured corticospinal motor neurons (CSMNs) purified from young animals[12]. Thus, it appears that for both peripheral nervous system (PNS) and CNS neurons, responses to neurotrophins and other growth factors are critical for axon growth during development.

## Mechanisms for development-dependent decrease of axon growth ability

Despite the progress made in axon growth during development, little is known about what accounts for the transition from the rapid growth mode of immature neurons into the poor growth mode of mature neurons in the CNS. Several potential players have been implicated, such as development-dependent decline of neuronal cAMP levels[13], or Bcl-2[14]. By analyzing the axon growth rates of RGCs, Goldberg et al[15] found a dramatic decrease occurring at the neonatal stage and this might be triggered by signal(s) from amacrine cells. However, the molecular nature of such signal(s) still remains elusive. In addition, anaphase promoting complex (APC), a protein complex with E3 ubiquitin ligase having a well-documented function in cell cycle control, has been recently implicated in controlling axon growth ability[16]. Some experimental evidence implicated that the APC activity remains active in post-mitotic neurons, and it may degrade proteins required for axon growth, such as the transcription factors ID2 and SnoN[17],[18]. However, whether these molecules are relevant to the axon regenerative ability *in vivo* is unclear.

## Phosphatase and tensin homolog/rapamycin (PTEN/mTOR) pathway in axon growth control

**Fig.1:**
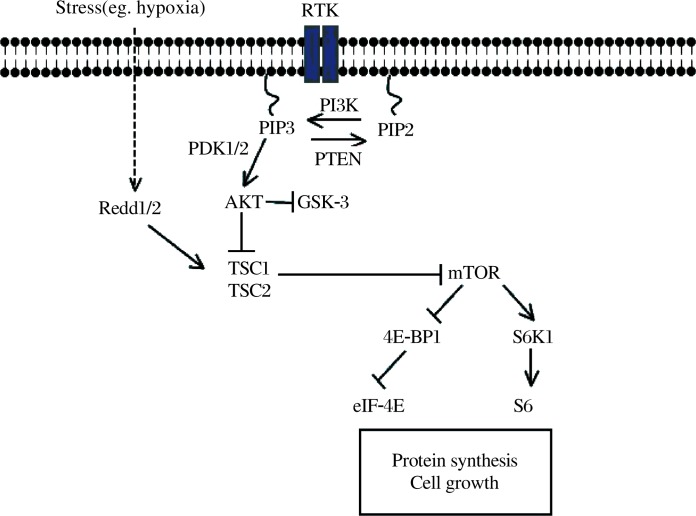
Scheme of the PTEN/mTOR signaling pathway. In response to receptor tyrosine kinase (RTK) activation, PI3K phosphorylates and converts the lipid second messenger PIP_2_ into PIP_3_, which recruits and activates phosphatidylinositol-dependent kinase 1/2 (PDK1/2). PDK1/2, in turn, phosphorylates and activates Akt. PTEN catalyzes the conversion from PIP_3_ to PIP_2_. Thus, inactivation of PTEN results in the accumulation of PIP_3_ and the activation of Akt. Akt controls a host of signaling molecules, including TSC1/2. Downstream of the TSC1/2 complex lies mTOR, which integrates various cellular signals, including nutrient availability to control protein translation, cell growth, and other processes. The ribosomal protein S6 kinase (RP-S6) and the eukaryotic initiation factor 4E (eIF-4E) binding protein 1 (4E-BP1) are the mTOR effector molecules executing these functions. Cellular stresses such as hypoxia induce expression of Redd1/2, which augments TSC1/2 activity and in turn suppress the mTOR activity.

Development-dependent decline of axon growth ability is reminiscent of cell size control in almost any cell type: active growth during development followed by ceased growth upon the completion of development result in a normally fixed size for individual cell types. Extensive studies in the fields of developmental biology and cancer biology have identified a number of genes critical for regulating cellular growth and many of these are tumor suppressor genes. Because many of these growth-control molecules are expressed in the adult neurons, we hypothesized that the mechanisms preventing individual cell types from over-growth might also play a role in suppressing the axon growth ability of adult neurons. To test this, we utilized an optic nerve crush model to examine the regeneration of axons from RGCs in different mutant mice with the deletion of individual growth control genes in the RGCs. By analyzing more than 10 different conditional deletion mouse lines, we found that deletion of PTEN promotes both the survival of axotomized RGCs and the robust regeneration of injured optic nerve fibers[1]. PTEN is a critical intracellular regulator of cellular responses to growth factors (***Fig. 1***, ref. 19-21). It catalyzes the conversion from phosphatidylinositol (3,4,5) trisphosphate (PIP_3_) to phosphatidylinositol (4,5) bisphosphate (PIP_2_) and antagonizes the effects of PI3K. Thus, inactivation of PTEN results in the accumulation of PIP_3_ and subsequently the activation of the Akt. A well characterized downstream event of PTEN deletion and Akt activation is the activation of mTOR, which is a central regulator of cap-dependent protein translation initiation and cell growth[19]–[21]. Thus, it is possible that PTEN deletion results in enhanced mTOR activity and new protein synthesis which may sustain axon regrowth for regeneration. Importantly, our studies revealed that mTOR activity is significantly down-regulated in injured CNS neurons, but not in PNS neurons[1]. Thus, the lack of mTOR activity may represent a major intrinsic obstacle for axon regeneration in injured CNS neurons. These studies strongly support the hypothesis that the activity of the PTEN/mTOR pathway is a key determinant of the intrinsic axon growth ability of adult CNS neurons. It will be important to assess whether manipulating this pathway could promote axon regeneration from other types of CNS neurons and whether these regenerating axons are able to form functional synapses.
